# Cannabidiol interacts with the FXR/Nrf2 pathway and changes the CB1/CB2 receptors ratio in gentamicin-induced kidney injury in rats

**DOI:** 10.22038/IJBMS.2023.67998.14867

**Published:** 2023-03

**Authors:** Vahideh Hokmabadi, Azadeh Khalili, Seyed Ali Hashemi, Keshvad Hedayatyanfard, Soraya Parvari, Saeed Changizi-Ashtiyani, Gholamreza Bayat

**Affiliations:** 1 Department of Physiology, School of Medicine, Arak University of Medical Sciences, Arak, Iran; 2 Department of Physiology, Pharmacology, Medical Physics, School of Medicine, Alborz University of Medical Sciences, Karaj, Iran; 3 Department of Pathology, School of Medicine, Alborz University of Medical Sciences, Karaj, Iran; 4 Department of Anatomical Sciences, School of Medicine, Alborz University of Medical Sciences, Karaj, Iran; # These authors contributed eqully to this work

**Keywords:** Cannabidiol, CB1 Receptors, CB2 Receptors, Farnesoid receptors, Gentamicin, Nrf2

## Abstract

**Objective(s):**

Gentamicin-induced nephrotoxicity was used as an experimental model of kidney disease. The present study was performed to assess the therapeutic role of cannabidiol (CBD) against gentamicin-induced renal damage.

**Materials and Methods:**

Forty two male Wistar rats were randomly allocated into 6 groups (n=7), including: (1) Control, (2) Vehicle, (3) Gentamicin-treated group (100 mg/kg/day) for 10 days (GM), (4-6) 3 Gentamicin-CBD-treated groups (2.5, 5, and 10 mg/kg/day) for 10 days (GM+CBD2.5, GM+CBD5, GM+CBD10). Serum levels of BUN and Cr, renal histology as well as real-time qRT-PCR were used to investigate the pattern of changes at different levels.

**Results:**

Gentamicin increased serum BUN and Cr (*P*<0.001), down-regulation of FXR (*P*<0.001), SOD (*P*<0.05) and up-regulation of CB1 receptor mRNA (*P*<0.01). Compared to the control group, CBD at 5 decreased (*P*<0.05) and at 10 mg/kg/day increased the expression of FXR (*P*<0.05). Nrf2 expression in CBD groups was increased (*P*<0.001 vs. GM). The expression of TNF-α compared to the control and GM groups, was significantly increased in CBD2.5 (*P*<0.01) and CBD10 (*P*<0.05). Compared to the control, CBD at 2.5 (*P*<0.01), 5 (*P*<0.001) and 10 (*P*<0.001) mg/kg/day significantly increased the expression of CB1R. Up-regulation of CB1R in the GM+CBD5, was significantly higher (*P*<0.05) than the GM group. Compared to the control group, the most significant increase in CB2 receptor expression was observed at CBD10 (*P*<0.05).

**Conclusion:**

CBD particularly at 10 mg/kg/day might be of significant therapeutic benefit against such renal complications. Activating the FXR/Nrf2 pathway and counteracting the deleterious effects of CB1 receptors via CB2 receptors scale-up could be part of the protective mechanisms of CBD.

## Introduction

It is well known that acute kidney injury (AKI) is associated with reduced glomerular infiltration rate, oliguria or anuria, electrolyte imbalance, increased serum creatinine, and finally, loss of function of the kidney which induces morbidity and mortality (1-3). It seems that the rate of incidence of AKI is increasing in developing countries.

The pathophysiology of AKI is not well understood but there are several risk factors including chronic hypertension, heart failure, and/or medications (4, 5). Some drugs such as aminoglycosides (neomycin, amikacin, and gentamicin), vancomycin, colistin, amphotericin B, antiviral drugs (cidofovir, adefovir), cisplatin, cyclosporine, and tacrolimus, may unexpectedly increase the risk of AKI (4). Some serious conditions including older age, diabetes, systemic disease, duration of therapy, and dosage may affect drug-induced nephrotoxicity. Various researchers have studied the role of inflammation and cytokines, macrophage, and neutrophil cells in the necrosis of kidney tissue and the development of AKI (2).

Gentamicin (GM) is one of the most important antibiotics used for the treatment of bacterial infections. Gentamicin induces nephrotoxicity and the use of this drug should be considered in prolonged therapy. Previous studies showed that GM increased the tubular necrosis. Furthermore, gentamicin elevated the inflammatory cells, proinflammatory cytokines (Tumor necrosis factor alpha, TNF-α), Transforming growth factor β (TGF-β), increased lipid and protein oxidation, reactive oxygen species (ROS), and nuclear factor kappa B (NFkB) signaling pathways (1, 6).

Cannabidiol (CBD) is one of the major active metabolites of the* Cannabis sativa* plant. CBD has potent anti-inflammatory and antioxidant properties but has no psychoactive or euphoric effects. The pharmacological effects of CBD are mediated via two receptors: cannabinoid receptor 1 (CB1R) and cannabinoid receptor 2 (CB2R). Besides, the distribution and expression of these two receptors are different (7, 8).

Some reports indicate that CBD reduced the proinflammatory cytokines (TNF-α and IL-1β) as well as ameliorated lipid and protein oxidative damage in the renal ischemic model (9). Another study showed that CBD decreased oxidative stress and inflammation in cisplatin-induced nephrotoxicity in the mice model (10). Overexpression of CB1R (11) as well FXR down-regulation (12) could induce oxidative stress signals as well as inflammatory cascades. The beneficial effects of CB1R inhibition and/or CB2R activation have been considered as some of the effective therapeutic targets to protect against damages induced by CB1-CB2 receptor imbalance.

Despite several basic studies on the pharmacological functions of some selective CB1/CB2 receptor agonists and/or antagonists on experimental models of kidney disease, few reports are indicating the effects of CBD on acute and chronic kidney disease. This study aimed to investigate the protective effects of CBD on gentamicin-induced nephrotoxicity in a rat model and evaluate the expression of FXR, Nrf2, SOD, TNF-α, CB1R and CB2R mRNA. 

## Materials and Methods

***Chemicals***

CBD was purchased from Yunnan Hansu Biotechnology Co., Ltd (China). Ketamine and Xylazine were obtained from Alfasan (Woerden, Holland). 

***Animals***

Eight-week-old male Wistar rats were obtained from Royan Animal Breeding Center, Karaj, Iran. Animals were kept under standard conditions (12 hr light/dark cycle at 20–24 °C and 50±5% relative humidity). They had free access to food and water during the study. The animal care, experimentation, and procedures were performed according to the national guidelines and protocols approved by the Research Ethics Committee of Arak University of Medical Sciences (IR.ARAKMU.REC.1400.100) in accordance with the National Institute of Health Guide for the Care and Use of Laboratory Animals (NIH Publication No.85-23, revised 1996). 

***Experimental design and protocol***

Forty-two male Wistar rats (250±30 g) were randomly allocated into 6 experimental groups (n=7), including: (1) Control group, (2) Vehicle group, (3) Gentamicin-treated group receiving gentamicin for 10 consecutive days (GM), (4, 5, 6) Three Gentamicin-CBD-treated groups receiving CBD at 2.5, 5, and 10 mg/kg/day for 10 consecutive days (GM+CBD2.5, GM+CBD5, and GM+CBD10). CBD powder was dissolved in ethanol, Tween-20, and distilled water (1:1:8). All gentamicin groups received daily intraperitoneal injections of gentamicin at 100 mg/kg for 10 days. At the end of each respective time, animals were deeply anesthetized with intraperitoneal injections of ketamine (60 mg/kg) and xylazine (8 mg/kg). Blood samples were collected from the right ventricle and the kidney’s tissue was picked up for histological and molecular studies. All samples were stored at -80 °C. 

***Biochemical parameters***

At the end of the experiment, blood samples were gently collected from the right ventricle to determine the serum levels of Blood Urea Nitrogen (BUN) and Creatinine (Cr) using Pars Azmoon commercial kits (Pars Azmoon Co, INC, Karaj, Iran), according to the manufacturer’s guidelines.

***Histological assessments***

After scarifying, the left kidney was removed and immediately fixed in a 10% formalin solution. After dehydration and clearance, samples were embedded in paraffin wax. The paraffin blocks then were cut into sections of 5 µm thickness. Tissue staining of hematoxylin and eosin (H&E), Masson's trichrome, and Periodic acid shift (PAS) were performed for detecting any pathological signs of damage, fibrotic scars, or brush border loss.

***Quantitative real-time-RT PCR assessment***

To identify the mRNA expression of renal FXR, Nrf2, SOD, TNF-α, CB1R, and CB2R were performed using real-time RT-PCR according to protocol (13). Briefly, about 50 mg of the left kidney tissue was homogenized using a polytron tissue homogenizer (DAIHAN-brand Homogenizing Stirrer, HS-30E, Korea). The RNA then was extracted using Trizol (Yekta Tajhiz Azma Co Iran) based on the manufacturer’s instructions. Then the cDNA synthesis was performed using a Reverse Transcriptase cDNA synthesis kit (Yekta Tajhiz Azma Co Iran), based on the protocol. Expression of the aforementioned genes was measured by Real-Time PCR using SYBR Green (Yekta Tajhiz Azma Co Iran). Experiments were performed in duplicates as follows: denaturation at 95 °C for 10 min subsequently followed by 45 cycles at 95 °C for 10 sec and 60 °C for 10 sec and 72 °C for 10 sec. The expression level was normalized to the GAPDH and expressed as fold-change. The exact nucleotide sequences of the genes and GAPDH primers were shown in Table 1. 

***Statistical analysis***

Quantitative data was presented as Mean±SEM. One way analysis of variance (ANOVA) was used for between-group analysis which then followed by Tukey’s test if any differences was detected. Non-quantitative data analysis was performed with Kruskal-Wallis nonparametric test and presented as the median (IQR). *P-*value of less than 0.05 was considered statistically significant. Graphpad Prism 8 (8.0.2) was used to analyze data and draw the graphs.

## Results

***Animal’s body and kidney weight***

The values of initial and final body weight as well as the mean difference between the two weights were summarized in Table 2. There were no significant differences between the initial and final body weights in the control and vehicle groups. But compared with the control group, the same values were significantly lower in all groups of GM (*P*<0.001), GM+CBD2.5 (*P*<0.001), GM+CBD5 (*P*<0.001), and GM+CBD10 (*P*<0.001). There was no significant difference between the aforementioned groups.

Left and right kidney weights as either absolute value or ratio to body weights are summarized in Table 3. The values between the control and vehicle groups were not statistically different. compared with the control group, the absolute weights of either left or right kidneys did not change during 10-day treatment with GM and GM+CBD, whereas, the ratio to body weights was significantly increased in all groups of GM (*P*<0.01, *P*<0.01), GM+CBD2.5 (*P*<0.001, *P*<0.001), GM+CBD5 (*P*<0.05, *P*<0.01), and GM+CBD10 (*P*<0.001, *P*<0.001), respectively. There was no significant difference among the aforementioned groups.

***Serum BUN and creatinine levels***

**Figure 1:**
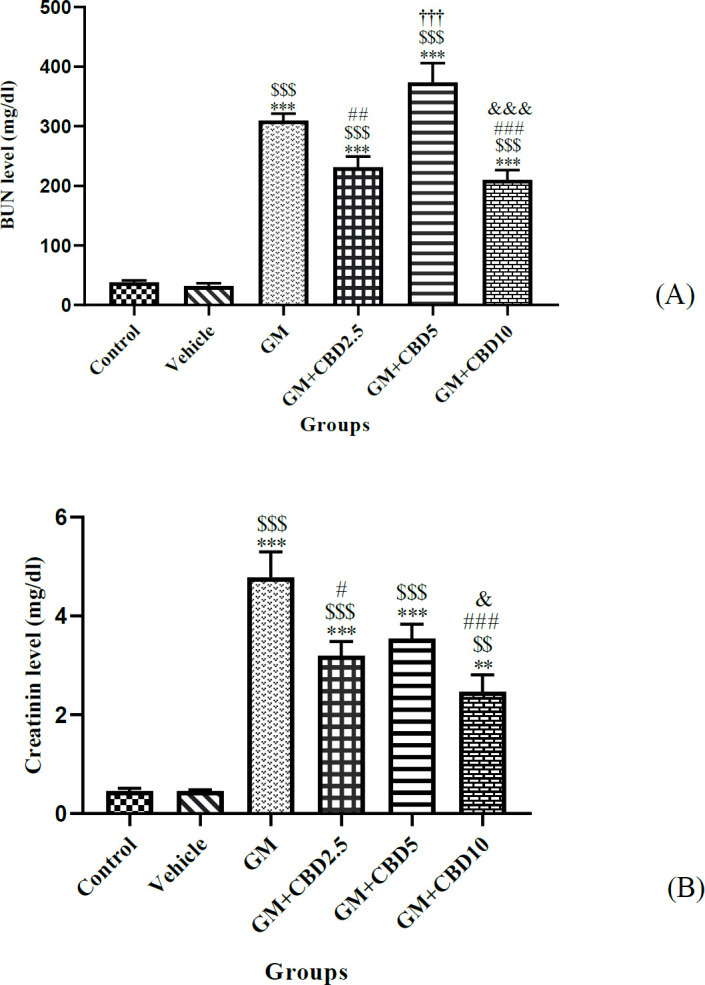
Effect of gentamicin (GM) and Gentamicin + Cannabidiol (CBD) administration on serum levels of (A) blood urea nitrogen (BUN) and (B) Creatinine (Cr) for 10 days. Data were presented as mean ± SEM in the experimental groups (n=7) including the control group, vehicle group, GM (100 mg/kg) group, GM+CBD2.5 (2.5 mg/kg) group, GM+CBD5 (5 mg/kg) group and GM+CBD10 (10 mg/kg) group for 10 days

As summarized in Figures 1A (BUN) and B (Cr), there was no significant difference in the serum levels of either BUN or Cr between the control and vehicle groups. However, compared with the control group, the values of BUN (*P*<0.001) and Cr (*P*<0.001) were significantly increased in the GM-treated group. 

The values of BUN and Cr in CBD-treated groups of 2.5 (*P*<0.001, *P*<0.001), 5 (*P*<0.001, *P*<0.001), and 10 (*P*<0.001, *P*<0.01) mg/kg/day, were still significantly higher than the control ones, respectively. When compared with the GM group, treatment with CBD at 2.5 (*P*<0.01, *P*<0.05) and 10 (*P*<0.001, *P*<0.001) mg/kg/day significantly decreased either BUN or Cr levels, respectively. The value of BUN in GM+CBD5 was significantly higher compared with 2.5 (*P*<0.001) and 10 (*P*<0.001) mg/kg/day. Moreover, the serum Cr level of GM+CBD10 was significantly lower than that of the GM+CBD5 value (*P*<0.05). 

***Histological assessment***

*Effects of GM and GM+CBD administration on glomerular and cortical tubular tissue*

**Figure 2:**
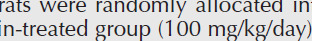
Histological changes of rat left kidney following Gentamicin (GM), Gentamicin + Cannabidiol (CBD) administration. Light Photomicrographs of histological sections of the cortex focused on glomeruli parts (H&E staining; magnification ×400) from experimental groups (n=7) of the control group (a), vehicle group (b), GM (100 mg/kg) group (c), GM+CBD2.5 (2.5 mg/kg) group (d), GM+CBD5 (5 mg/kg) group (e) and GM+CBD10 (10 mg/kg) group (f)

**Figure 3:**
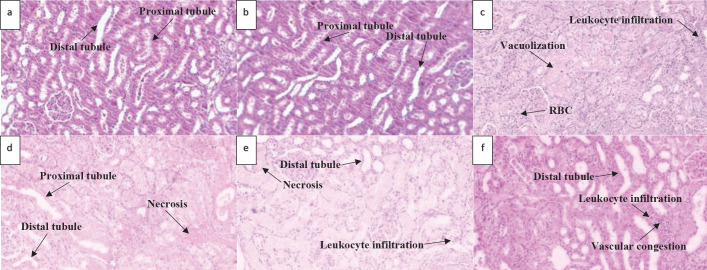
Histological changes of rat left kidney following Gentamicin (GM), Gentamicin + Cannabidiol (CBD) administration. Light Photomicrographs of histological sections of the cortex focused on tubular parts (H&E staining; magnification ×400) from experimental groups (n=7) of the control group (a), vehicle group (b), GM (100 mg/kg) group (c), GM+CBD2.5 (2.5 mg/kg) group (d), GM+CBD5 (5 mg/kg) group (e) and GM+CBD10 (10 mg/kg) group (f)

**Figure 4:**
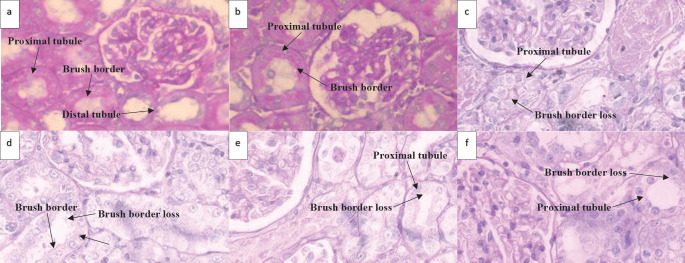
Histological changes of rat left kidney following Gentamicin (GM), Gentamicin + Cannabidiol (CBD) administration. Light Photomicrographs of histological sections of the cortex focused on proximal tubule brush borders (PAS staining; magnification ×1000) from experimental groups (n=7) of the control group (a), vehicle group (b), GM (100 mg/kg) group (c), GM+CBD2.5 (2.5 mg/kg) group (d), GM+CBD5 (5 mg/kg) group (e) and GM+CBD10 (10 mg/kg) group (f)

**Figure 5:**
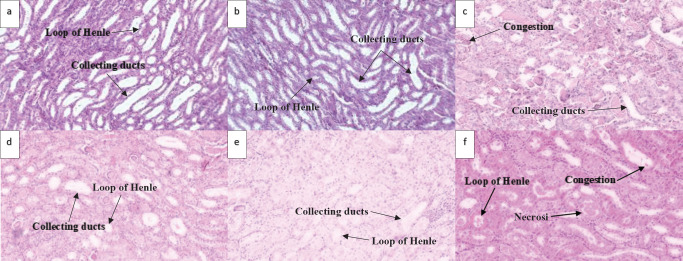
Histological changes of rat left kidney following Gentamicin (GM), Gentamicin + Cannabidiol (CBD) administration. Light Photomicrographs of histological sections of the outer medulla (H&E staining; magnification ×400) from experimental groups (n=7) of the control group (a), vehicle group (b), GM (100 mg/kg) group (c), GM+CBD2.5 (2.5 mg/kg) group (d), GM+CBD5 (5 mg/kg) group (e) and GM+CBD10 (10 mg/kg) group (f)

**Figure 6.**
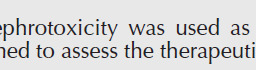
Histological changes of rat left kidney following Gentamicin (GM), Gentamicin + Cannabidiol (CBD) administration. Light Photomicrographs of histological sections of the outer medulla (PAS staining; magnification ×400) from experimental groups (n=7) of the control group (a), vehicle group (b), GM (100 mg/kg) group (c), GM+CBD2.5 (2.5 mg/kg) group (d), GM+CBD5 (5 mg/kg) group (e) and GM+CBD10 (10 mg/kg) group (f)

Changes in the cortical and outer medullary sections have been shown in Table 4 and Figure 2-6. As shown in H&E staining of cortical parts (Figure 2), compared with that of the control group, 10 days of administration of GM was associated with a significant increase in glomerular size, the space of the bowman’s capsules, the number of RBCs in the luminal capillaries as well as the infiltration of the polymorphonuclear (PMN) leukocytes. Interestingly, 10 days of treatment with the lowest (2.5 mg/kg/day) and the highest (10 mg/kg/day) doses of CBD were associated with the marked correction of aforementioned cortical disarrangement signs. In contrast, the median dose (5 mg/kg/day) could not repair such glomerular damage (Table 4).

As shown in Table 4 and Figure 3, H&E staining showed that compared with the control ones, cortical tubular damages were significantly developed during 10 days of GM administration. Such cortical damages were characterized by significant leukocyte infiltration, vacuolization, cell desquamation, and epithelial cell necrosis. Treatment with CBD2.5 and to a lesser extent CBD10 was effective in rearrangement of such tubular damages (Table 4).

As exhibited in Table 4 fibrotic bundles were not seen with Massonʼs trichrome staining in any of the experimental groups.

Detection of brush border loss was also confirmed by GM-induced cortical damage (Table 4, Figure 4). Although such damage still remained during CBD administration, the median dose (5 mg/kg/day) was associated with the highest degree of brush border loss.

*Effects of GM and GM+CBD administration on outer medullary tissue*

Histological assessment of outer medullary tubular sections (Table 4, Figure 5 and 6) showed significant signs of vascular congestion, development of protein casts, cell desquamation, cytoplasmic vacuolization and epithelial necrosis in GM and GM+CBD5, compared with that of the control. Such histological tubular damages were significantly restored with CBD2.5 and CBD10.

***Molecular findings***

*Effects of GM administration on the expression of renal FXR, Nrf2, SOD, TNF-α, CB1R, and CB2R mRNA*

**Figure 7:**
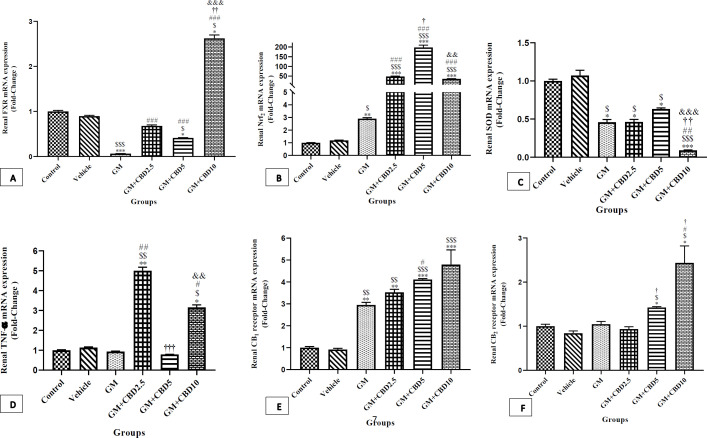
Effect of Gentamicin (GM), Gentamicin + Cannabidiol (CBD) administration on the renal expression of targeted genes in a 10 days. Data were presented as mean ± SEM in the experimental groups (n=7) including the control group, vehicle group, GM (100 mg/kg) group, GM+CBD2.5 (2.5 mg/kg) group, GM+CBD5 (5 mg/kg) group and GM+CBD10 (10 mg/kg) group

Findings of changes in the mRNA gene expressions have been shown in Figure 7A-F. 

The expression of genes was the same between the control and vehicle groups. Compared with the control group, 10 days of administration of GM was associated with a significant decrease in the expression of FXR (*P*<0.001), and SOD (*P*<0.05), and an increase in Nrf2 (*P*<0.01), and CB1R (*P*<0.01). The expression of TNF-α and CB2R did not change due to GM administration alone.

*Effects of GM+CBD administration on the expression of renal FXR, Nrf2, SOD, TNF-α, CB1R, and CB2R mRNA*

As shown in Figure 7A-F, CBD administration could affect the expression of the aforementioned genes. Compared with the control group, administration of CBD at 2.5mg/kg did not change the expression of FXR, whereas CBD at 5 and 10mg/kg were associated with significant down-regulation (*P*<0.05), and up-regulation (*P*<0.05), respectively. Generally, regardless of between-group differences and compared with the GM group, the expression of FXR (*P*<0.001 for each) in all CBD-treated groups, was significantly increased (Figures 7A). Compared with the control group, administration of CBD in all treated groups were associated with significant Nrf2 up-regulation (*P*<0.001 for each) (Figure 7B). 

The same trend was not observed for other genes. Compared with the control group, SOD mRNA expression was significantly decreased due to administration of CBD at 2.5 (*P*<0.05), 5 (*P*<0.05), and 10 (*P*<0.001) mg/kg. In contrast to our expectation, the highest down-regulation of SOD mRNA expression was seen in GM+CBD10 group (Figure 7C) so that, there was a significant difference with the GM (*P*<0.01), CBD2.5 (*P*<0.01), and CBD5 (*P*<0.001). 

In comparison with the Control and GM groups, administration of CBD at 2.5 (*P*<0.01, *P*<0.01) and CBD10 (*P*<0.05, *P*<0.05) mg/kg, increased the expression of TNF-α (Figure 7D), respectively. The value in the CBD5 was the same as the control one.

In comparison with the control group, the expression of CB1R at 2.5 (*P*<0.01), 5 (*P*<0.001), and 10 (*P*<0.001) mg/kg of CBD was associated with significant up-regulation, however, compared with the GM group, treatment with CBD at 2.5 and 10 mg/kg/day did not change the expression of CB1R. The expression of CB1R in the CBD5 group, was significantly higher (*P*<0.05) than the GM one (Figure 7E).

Similar to the GM group, CBD2.5 did not show any significant change in the mRNA expression of CB2R, so the value was as same as the control group (Figure 7F). In contrast, the mRNA expression of the gene was associated with marked up-regulation in either CBD5 (*P*<0.05) or CBD10 (*P*<0.05). Compared with the GM group, the expression of CB2R was significantly increased with CBD at 10 mg/kg (*P*<0.05).

**Table 1:** Nucleotide sequences of primers (Rat) used for quantitative real-time RT-PCR

Primer sequences (5´-3´)		Genes
Reverse	Forward	
CCTGTGGCATTCTCTGTTTG	**TGGGAATGTTGGCTGAATG**	FXR
GGGAATGTCTCTGCCAAAAG	**AGTGGATCTGTCAGCTACTC**	Nrf2
TCCAGCATTTCCAGTCTTTG	**TGTGTCCATTGAAGATCGTG**	SOD
TGATCTGAGTGTGAGGGTC	**TCTGTCTACTGAACTTCGGG**	TNF-α
TGCCGATGAAGTGGTAGGAAG	**CGTCGTTCAAGGAGAATGAGG**	CB1
GGGGCTTCTTCTTTCCCCTC	**GAGTGGAGAGATCCGCTCTG**	CB2
CTTCCCATTCTCAGCCTT	GCCTTCTCTTGTGACAAAGTG	GAPDH

**Table 2:** Effect of gentamicin and gentamicin+ Cannabidiol (CBD) administration on animal’s body weights

Groups	Average initial weight (g)	Average final weight (g)	Average weight difference (g)
Control	**266±7.97**	**298±7.37**	**32±1.58**
Vehicle	**269±9.41**	**279.6±13.03**	**10.6±5.01**
GM	**264.2±5.59**	**229±6.89** ^***$$^	**-35.17±5.24** ^***$$$^
GM+CBD2.5	**271±5.97**	**224.4±6.84** ^***$$^	**-46.57±5.09** ^***$$$^
GM+CBD5	**267.1±8.65**	**220±7.90** ^***$$$^	**-47.14±7.26** ^***$$$^
GM+CBD10	**271.7±8.22**	**237.2±8.96** ^***$^	**-34.5±2.31** ^***$$$^

Data were presented as mean±SEM in the experimental groups (n=7) including the control group, vehicle group, gentamicin (GM, 100 mg/kg) group, GM+CBD2.5 (2.5 mg/kg) group, GM+CBD5 (5 mg/kg) group and GM+CBD10 (10 mg/kg) group for 10 days

***P<0.001 vs Control group, ^$^*P*<0.05, ^$$^*P*<0.01, and ^$$$^*P*<0.001 vs Vehicle group

**Table 3:** Effect of gentamicin and gentamicin+ Cannabidiol (CBD) administration on animal kidneys weights

Groups	Average right kidney weight (g)	Average right kidney weight (mg) to body weight ratio (g)	Average left kidney weight (g)	Average left kidney weight (mg) to body weight ratio (g)
Control	**1.080±0.022**	**3.640±0.102**	**1.080±0.037**	**3.630±0.107**
Vehicle	**0.943±0.049**	**3.370±0.055**	**0.899±0.043**	**3.220±0.101**
GM	**1.100±0.036**	**4.800±0.133** ^**$$$^	**1.070±0.046**	**4.660±0.149** ^**$$$^
GM+CBD2.5	**1.160±0.070**	**5.170±0.262** ^***$$$^	**1.130±0.065**	**5.030±0.241** ^***$$$^
GM+CBD5	**1.010±0.055**	**4.590±0.186** ^*$$^	**0.995±0.030**	**4.540±0.116** ^**$$$^
GM+CBD10	**1.180±0.051**	**5.000±0.204** ^***$$$^	**1.170±0.048**	**4.950±0.183** ^***$$$^

Data were presented as mean±SEM in the experimental groups (n=7), including the control group, vehicle group, gentamicin (GM, 100 mg/kg) group, GM+CBD2.5 (2.5 mg/kg) group, GM+CBD5 (5 mg/kg) group, and GM+CBD10 (10 mg/kg) group for 10 days

* *P*<0.05, ** *P*<0.01, *** *P*<0.001 vs Control group, ^$$^ *P*<0.01, ^$$$^ *P*<0.001 vs Vehicle group

** *P*<0.01 and *** *P*<0.001 vs Control group; ^$$^ *P*<0.01 and ^$$$^ *P*<0.001 vs Vehicle group; ^# ^*P*<0.05,^ ##^ *P*<0.01 and ^###^ *P*<0.001 vs GM group; ^†††^ *P*<0.001 vs GM+CBD2.5 group; ^& ^*P*<0.05 and ^&&&^ *P*<0.001 vs GM+CBD5 group

**Table 4:** Effect of gentamicin and gentamicin+ Cannabidiol (CBD) administration on renal histology

H&E staining		Groups					
Cortex: Glomerulus		Control	Vehicle	GM	GM+CBD2.5	GM+CBD5	GM+CBD510
**1**	Size of glomeruli	0(0)	0(0)	↑2(1)^***$$$^	↑1(1)	↑1(0)	↑1(1)
**2**	Bowman’s space size	0(0)	0(0)	↑1(1.25)^*$ ^	↑1(1)	↑1(1)^*$^	↑1(1.25)
**3**	No. of RBC in the capillary lumen	0(0)	0(0)	↑2(0.5)^**$$^	↑1(0)	↑2(1)^*$^	↑1.5(1)^*$^
**4**	No. of mesangial cell	0(0)	0(0)	↑1(1)	↑1(1)	↑1(0)^**$$^	↑1(1)
**5**	PMN infiltration	0(0)	0(0)	1(0)^*$^	1(0)^*$^	1(0)^**$$^	1(1)
**Cortex: ** **Tubules**	**PT, TAL, DT, CCD**						
**1**	Leukocyte infiltration	0(0)	0(0)	2.5(2)^*$^	2(0)	2(1)^*$^	3(1)^**$$^
**2**	Desquamation	0(0)	0(0)	1.5(1)^*$^	1(1)^*$^	2(0)^***$$$^	1.5(1)^*$^
**3**	Epithelial cytoplasmic cell vacuolization	0(0)	0(0)	2(1)	2(1)^**$$^	2(0)^*$^	2.5(1.25)^**$$^
**4**	tubular epithelial cell necrosis	0(0)	0(0)	2(0.25)^**$$^	1(0)	2(0)^***$$$^	1.5(1)^*$^
**5**	Tubular regeneration	0(0)	0(0)	0(0.25)	0(1)	0(1)	1(0.25)^*$^
**Outer medulla**	**PST (S3),** **TDL, TAL, MCD**						
**1**	Vascular congestion	0(0)	0(0)	2(2)^**$$^	1(1)	2(1)^**$$^	1(1)
**2**	Intratubular proteinaceous casts	0(0)	0(0)	2.5(1)^***$$$^	2(1)^*$^	1(2)^*$^	1(1.25)
**3**	Desquamation	0(0)	0(0)	1(1)	1(1)	1(0)^**$$^	1(1.25)
**4**	Epithelial cytoplasmic cell vacuolization	0(0)	0(0)	1(0.25)^**$$^	1(0)^*$^	1(0)^**$$^	1(0)^**$$^
**5**	Tubular epithelial cell necrosis	0(0)	0(0)	1(0)^**$$^	1(1)	1(0)^**$$^	1(0)
**PAS staining**							
**1**	Brush border loss	0(0)	0(0)	3(1)^**$$^	3(0)^*$^	3(1)^***$$$^	3(0)^*$^
**2**	Thickening of the glomerular membrane	0(0)	0(0)	1(0.25)^*$^	1(1)	1(0)^**$$^	0(0.25)^&^
**Mason’trichrome staining**							
**1**	Fibrotic bundle	0(0)	0(0)	0(0)	0(0)	0(0)	0(0)

Data were presented as median (IQR) in the experimental groups (n=7) including the control group, vehicle group, gentamicin (GM, 100mg/kg) group, GM+CBD2.5 (2.5 mg/kg) group, GM+CBD5 (5 mg/kg) group and GM+CBD10 (10 mg/kg) group for 10 days

0: no abnormality detected; 1: damage/active changes up to <20%; (Mild) 2: damage/active changes up to 20 - 40% (Moderate); 3: damage/active changes up to 40- 60%; 4: damage/active changes up to-60-80%; 5: damage/active changes up to >80%. Polymorphonuclear=PMN, Proximal tubule=PT, Thick ascending limb=TAL, Distal tubule=DT, Cortical collecting duct=CCD, Proximal straight tubule=PST (S3), Thin descending limb=TDL, Thick ascending limb=TAL, Medullary collecting duct=MCD

* *P*<0.05, ** *P*<0.01, *** *P*<0.001 vs Control group, ^$^ *P*<0.05, ^$$^ *P*<0.01, ^$$$^ *P*<0.001 vs Vehicle group, ^&^ *P*<0.05 vs GM+CBD5 group

*P<0.05, **P<0.01, ***P<0.001 vs Control group; ^$^*P*<0.05, ^$$^*P*<0.01, ^$$$^*P*<0.001 vs Vehicle group;^ #^*P*<0.05, ^##^*P*<0.01, ^###^*P*<0.001 vs GM group; ^†^*P*<0.05, ^††^*P*<0.01, ^†††^*P*<0.001 vs GM+CBD2.5 group, ^&&^*P*<0.01 and ^&&&^*P*<0.001 vs GM+CBD5 group

## Discussion

The findings of the present study showed that administration of GM at 100 mg/kg for 10 consecutive days negatively affected either the histological structure or functional performance of the kidney. Such kidney impairment was also confirmed by molecular findings such as significant down-regulation of FXR and SOD mRNA as well as marked up-regulation of CB1R mRNA expression. 

The main objective of the present study was to evaluate the therapeutic potential of different doses of CBD against the destructive effects of GM on kidneys. According to our findings, CBD was effective in different aspects of the kidney’s performance but not in a dose-dependent behavior. Significant reduction in serum BUN and Cr marked improvement in cortical and outer medullary disarrangement signs induced by GM, along with no statistically significant change in the mRNA expression of CB1R, were the positive signs of the therapeutic potentials of the lowest and the highest doses of CBD. In spite of such therapeutic effects of CBD2.5 and 10 mg/kg/day, the median dose of 5 mg/kg/day was paradoxically associated with the progression of cortical and medullary damages and consequently the highest elevation of serum BUN and Cr. Moreover, significant up-regulation of CB1R was observed in CBD5, when compared with the GM one. 

Gentamicin-induced nephrotoxicity is a limiting issue that cause some clinical concerns during its administration. So, in the basic preclinical studies, the experimental model of GM-induced renal failure is used to discover some underlying pathological mechanisms of the disease and also to find some therapeutic candidates for such illness. Similar to previous reports (14, 15), the present study also showed massive histological damages either in cortical or outer medullary parts as well as marked elevation of BUN and Cr, as kidney function markers. According to others, such typical damage by GM comes from accumulation of the drug in the epithelial cells, especially in the proximal tubule (16). 

Several studies have evaluated the effect of the endocannabinoid system on renal disease. Up-regulation of CB1 receptors in some renal diseases such as the experimental model of renal fibrosis (11) and diabetic nephropathy in mice (17-19) along with down-regulation of CB2 receptors in diabetic nephropathy mice (20) suggest the pathologic role of CB1R up-regulation against the protective role of CB2 receptors. The new idea was also confirmed by pharmacological studies indicating that using CB1 receptor antagonists and/or CB2 receptor agonists could improve such renal impairments (11, 17). According to Barutta *et al*. (20) “Signaling through CB2 receptor on either inflammatory or parenchymal cells has anti-inflammatory effects and lowers inflammation-driven fibrosis, while signaling through CB1 receptor promotes oxidative stress and inflammation, resulting in both cell apoptosis and fibrosis”. 

Consistent with previous reports, in the present experiment, the expression of the CB1 receptor was up-regulated in the GM-treated group. It was also associated with a severe decline in the expression of FXR and SOD. It seems that there is a relationship between such molecular dysregulation and other biochemical and histological changes. In contrast, the expression of Nrf2 was not consistent with the FXR one. Nrf2 up-regulation might be an aggressive response to protect the cell against such SOD reduction. As previous studies have reported, overexpression of CB1R (11) besides FXR down-regulation (12) could induce oxidative stress signals as well as inflammatory cascades. 

The beneficial effects of CB1 receptor inhibition and/or CB2 receptor activation have been considered as some of the effective therapeutic targets to protect against damages induced by CB1-CB2 receptors imbalance. CBD, as a nonpsychoactive cannabis-derived compound possesses several pharmacological properties such as antianxiety, anti-inflammatory, analgesic, and anticonvulsant (21). CBD has a very low affinity for either CB1R or CB2R but it could antagonize the effects of CB1/CB2 receptor agonists. Indeed, it acts as a non-competitive allosteric antagonist on the cannabinoid receptors (21, 22). CBD also counteracts the activating effects of the psychoactive component of cannabis, tetrahydrocannabinol (THC), on CB1R via its negative allosteric modulator effect on the receptor (23). Despite several basic studies on the pharmacological functions of some selective CB1/CB2 receptor agonists and/or antagonists on experimental models of kidney disease, there are few reports indicating the effects of CBD on acute and chronic kidney disease. In the present study, GM-induced nephrotoxic symptoms, either biochemical or histologic ones, were obviously ameliorated by the lowest and the highest CBD doses. These two doses of 2.5 and particularly 10 mg/kg/day, were also associated with the highest extent of FXR up-regulation. Furthermore, despite CBD5, administration of CBD2.5 and CBD10 mg/kg/day did not induce CB1 receptor up-regulation, but CBD10 resulted in a significant CB2R up-regulation. These molecular alterations besides Nrf2 overexpression, particularly with CBD10, collectively could protect cells against such xenobiotic-generated oxidative stress. A similar renoprotective effect had been also confirmed by Pan *et al*. (10). They showed that IP injection of CBD at 10/mg/kg/day for 72 hr improved renal function and markedly attenuated the cisplatin-induced oxidative/nitrosative stress, inflammation, and cell death (10). In spite of Pan *et al*. findings which exhibited a dose-dependent relationship among CBD2.5, 5, and 10, for an unknown reason, in the present study, CBD5 showed deleterious effects on kidney function that were characterized by rising in BUN, Cr, and several cortical and outer medullary damages. Such nephrotoxic effects might be explained by significant up-regulation of CB1R mRNA. Although CB2R up-regulation was also observed for CBD5, it seems that it was not enough to counteract against deleterious effects of CB1R over-expression. 

## Conclusion

Administration of CBD at 2.5 and 10 mg/kg/day for 10 consecutive days, along with GM treatment, has improved renal pathological impairment and corrected several histological disarrangements. Regarding either histological or molecular findings, in this study, the most effective dose of CBD against GM-induced renal damage was 10 mg/kg/day. So it could be concluded that CBD particularly at 10 mg/kg/day might have therapeutic potential against GM-induced renal complications by activating the FXR/Nrf2 pathway and counteracting the deleterious effects of CB1 receptors by scaling up the CB2 ones.

## Authors’ Contributions

GB, SChA, AKh, VH, SAH, and KH conceived the study and design; GB, SChA, AKh, SAH, VH, KH, and SP performed experiments, data collection, analysis, and prepared the draft manuscript; GB, SChA, AKh, SAH, VH, KH, and SP critically revised and/or edited the paper; GB and SChA supervised the research. 

## Conflicts of Interest

The authors declare that they have no conflicts of interest. 
